# Understanding *Mycobacterium tuberculosis* through its genomic diversity and evolution

**DOI:** 10.1371/journal.ppat.1012956

**Published:** 2025-02-28

**Authors:** Mollie I. Sweeney, Carson E. Carranza, David M. Tobin

**Affiliations:** 1 Department of Molecular Genetics and Microbiology, Duke University School of Medicine, Durham, North Carolina, USA; 2 Department of Integrative Immunobiology, Duke University School of Medicine, Durham, North Carolina, USA; Tufts Univ School of Medicine, UNITED STATES OF AMERICA

## Abstract

Pathogen evolution and genomic diversity are shaped by specific host immune pressures and therapeutic interventions. Analysis of the extant genomes of circulating strains of *Mycobacterium tuberculosis*, a leading cause of infectious mortality that has co-evolved with humans for thousands of years, can provide new insights into host-pathogen interactions that underlie specific aspects of pathogenesis and onward transmission. With the explosion in the number of fully sequenced *M. tuberculosis* strains that are now paired with detailed clinical data, there are new opportunities to understand the evolutionary basis for and consequences of *M. tuberculosis* strain diversity. This review examines mechanistic findings that have emerged from pairing whole genome sequencing data and evolutionary analysis with functional dissection of specific bacterial variants. These include improved understanding of secreted effectors that modulate the properties and migratory behavior of infected macrophages as well as bacterial genetic alterations important for survival within hypoxic microenvironments. Genomic, evolutionary, and functional analyses across diverse *M. tuberculosis* strains will identify prominent bacterial adaptations to their human hosts and shape our understanding of TB disease biology and the host immune response.

## Introduction

**Fig 1:**
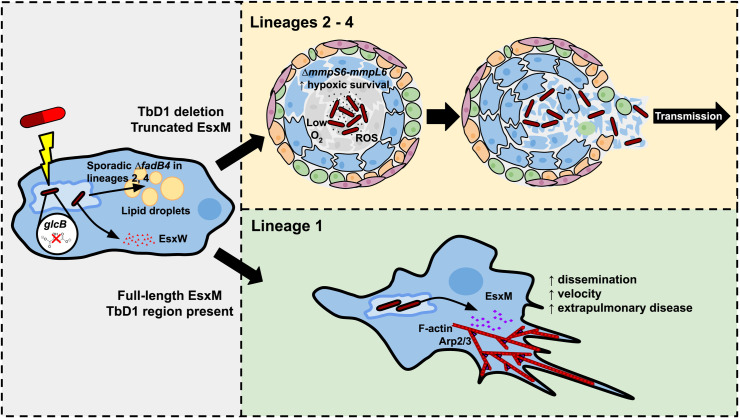
Insights into *Mycobacterium tuberculosis* pathogenesis from analysis of genetic diversity and evolution. Bacterial functional variants that have emerged from whole genome sequencing of diverse strains include genetic variation that influences resistance to antibiotics (for example, altered dependence of the *glcB* gene in sensitivity to a drug targeting the malate synthesis pathway) or the cell biology of infected macrophages. Deletions in bacterial *fadb4* from lineage 2 and 4 strains in the UK were associated with alterations in lipid droplet formation. Variants in secreted mycobacterial effectors that interact with host cells have also emerged, including in the gene encoding the secreted ESX-5 WXG protein EsxW, with one variant associated with increased transmission in Vietnam. At the major ESX-5 locus, *esxM* encodes a premature stop codon in virtually all lineage 2, 3, and 4 strains. In contrast, secretion of the full-length ancestral version – present in lineage 1 – leads to alterations in the actin cytoskeleton of infected macrophages, increases in the velocity and rates of migration of infected cells, and increased extrapulmonary dissemination. Similarly, a structural variant called TbD1 comprising deletion of *mmpS6*/*mmpL6* is implicated in increased bacterial survival during oxidative stress and hypoxia in lineages 2, 3, and 4. These variations and their combinations may contribute to differences in rates of extrapulmonary dissemination and transmission between and within lineages.

Tuberculosis disease (TB) continues to devastate populations worldwide, causing 10.8 million cases and 1.25 million deaths in 2023 [1]. TB is caused by the species *Mycobacterium tuberculosis,* but there is phenotypic and genomic diversity across strains. Genomic analysis has defined 10 discrete lineages of *M. tuberculosis* [2,3]. Given the absence of horizontal gene transfer in extant strains, deletions are almost always evolutionarily irreversible. For instance, a split between lineages that have historically been characterized as “ancestral” and those referred to as “modern” are marked by a region called TB deletion 1 (TbD1) that has been lost in the more geographically widespread lineages 2, 3, and 4 (Fig 1) [4]. The absence of these genes may be a driver of evolutionary success by altering the bacterial response to hypoxia within TB granulomas [5]. In the decades since the discovery of TbD1, our understanding of the genetic differences both within and between lineages has expanded with the application of next-generation sequencing across thousands of strains. Improved phenotypic data, including experimental, clinical and epidemiological characterization of many of these strains, may point to specific variants that influence pathogenesis and transmission. Evolutionary analysis of these datasets has also identified signatures of positive and negative selection throughout the *M. tuberculosis* genome [6]. This mini-review will highlight recent studies that have drawn connections between *M. tuberculosis* lineage-specific variants, transmissibility, and disease outcome, and discuss how investigating distinct *M. tuberculosis* strains has led to mechanistic insights into the success of *M. tuberculosis* as a pathogen. Deeper analysis of diverse *M. tuberculosis* strains [7] can enhance understanding of mycobacterial pathogenesis beyond what is possible through the investigation of standard laboratory strains alone.

## Experimental characterization of *M. tuberculosis* lineages

Basic research on diverse *M. tuberculosis* lineages has uncovered a variety of cellular processes that underlie different disease trajectories in humans. Although seminal contributions to our understanding of *M. tuberculosis* pathogenesis have been made at the bench using standard lineage 4 laboratory strains, recent studies utilizing strains representing a variety of *M. tuberculosis* lineages have identified previously unstudied mechanisms of bacterial survival and host evasion.

One issue of particular importance to public health is the frequent and varied drug resistance developed by *M. tuberculosis*. Although drug resistance to front-line antibiotics arises consistently in conserved targets across lineages, there is also emerging evidence that some resistance is a function of more complex interactions; resistance mediated by some variants may be lineage specific. Transposon mutagenesis-based screening and sequencing on a collection of 9 strains representing diverse lineages have identified differential genetic requirements for in vitro growth, which correspond to differences in drug sensitivity [8]. For example, strains from different lineages showed variable requirements for the malate synthase gene *glcB*; this dependence was reflected in the differential efficacy of a small molecule targeting GlcB (Fig 1) [8]. Another approach combining a CRISPRi drug screening platform with comparative genomics of clinical isolates identified an antibiotic resistance factor, *whiB7*, that appears to be inactivated in an entire L1 sub-lineage prevalent in southeast Asia [9].

Beyond antibiotic susceptibility, large-scale screening platforms can define lineage-dependent pathogen responses to physiologically relevant conditions. An in vitro study interrogating hypoxia resistance, an important survival mechanism for bacteria exposed to an oxygen-poor environment within a granuloma, found that L4 was uniquely able to recover from oxygen deprivation compared to L1-3 [10]. Although this study was not directly conducted in a host environment, it implies that hypoxia resistance may vary between lineages and sub-lineages. Such profiling of phenotypic diversity among *M. tuberculosis* lineages in vitro in conditions that mimic known in vivo microenvironments allows for screening of bacterial adaptations to host-like environments and identification of adaptation between and among strains.

Pairing clinical strains that can be studied experimentally with epidemiological and patient data has the power to identify mutations and characteristics of specific *M. tuberculosis* lineages that correlate with patient disease site and outcome. Utilizing barcoded isolates from a clinical cohort in Ho Chi Minh City encompassing lineage 1, 2, and 4 strains, Stanley et al. assessed bacterial fitness across a variety of metabolic states [11]. The authors not only found associations between slow in vitro growth and treatment failure but were also able to associate SNPs in a phosphodiesterase gene with treatment outcomes irrespective of *M. tuberculosis* lineage. While a full exploration of sub-lineage diversity is beyond the scope of this mini-review, large-scale analyses have successfully identified genetically unique sub-lineages associated with increased risk of specific disease presentations (e.g., cavitary disease and disseminated infections) as well as differences in inferred transmissibility [11,12].

A separate analysis of this cohort found that despite the endemic nature of L1 (Indo-Oceanic lineage) in Vietnam, the more recently introduced “modern” L2 Beijing lineage was more frequently transmitted, displacing transmission of the endemic L1 strain [13]. Computational screening for positive selection found that a mutation in EsxW, an effector within a canonical mycobacterial ESX secretion system important for pathogenesis, may play a role in the enhanced transmissibility of some strains in the region (Fig 1) [13]. Although the authors demonstrated a transmission advantage for a subset of circulating “modern” L2 strains, they also observed parallel evolution occurring in *esxW*, with the same mutation arising in L1, L2, and L4 strains [13]. Animal models of infection have uncovered other examples of lineage-specific differences. Comparison of the early infection behavior of a specific L2 strain versus the standard L4 lab strain in mice found more rapid spread from alveolar macrophages to recruited phagocytes in the lung as well as enhanced T cell activation [14]. A recently published open-access platform including over 50,000 *M. tuberculosis* genomes will serve as a useful tool for the exploration of inter- and intra-lineage genetic diversity, generating new hypotheses that can then be tested functionally [15].

## Insights from TB outbreak strains

Unique outbreak strains that may harbor mutations associated with clinical presentation and transmissibility provide another starting point for uncovering cellular and molecular mechanisms of pathogenesis. Originally identified as an outbreak strain with high transmission rates, HN878 is a hypervirulent L2 strain with a unique lipid profile marked by a phenolic glycolipid (PGL) that regulates the host immune response [16,17]. While such studies generally begin with a specific strain, genetic factors that are identified as drivers of various disease states often extend to other closely related strains or even entire lineages [16,17].

More recently, investigation of unique outbreak strains has led to the establishment of novel roles for mycobacterial factors and their impact on infection site. TB is transmitted from human lung to human lung, but bacteria can disseminate outside of the lung. Skeletal TB, while relatively rare in the United States, can cause devastating long-term damage. An outbreak with extraordinarily high rates of skeletal TB in North Carolina, USA, led to the discovery of a functional role for a small WXG-secreted effector encoded within the ESX-5 secretion system. The effector, EsxM, is truncated in lineages 2, 3, and 4 of *M. tuberculosis* (Fig 1). However, EsxM is encoded in its full length in “ancestral” lineages of *M. tuberculosis*, including the L1 outbreak strain, as well as in the other animal-adapted pathogenic mycobacterium species. Functional studies of EsxM in a zebrafish-*M. marinum* infection model and in human macrophages demonstrated that full-length EsxM promotes alterations in the migratory behavior of infected macrophages, promoting dissemination of mycobacterial infection [18]. Given the association between “ancestral” lineages and extrapulmonary TB, it is possible that the truncation of EsxM in “modern” lineages contributes to their relatively lower observed rates of dissemination and, perhaps, also influences rates of transmission [13,18–20].

Comparison of *M. tuberculosis* strains in populations exposed to both local, endemic strains and those introduced via migration have uncovered bacterial factors that may drive transmission dynamics and pathogen success. In one study in the U.K. midlands, researchers compared endemic *M. tuberculosis* outbreak strains with non-outbreak isolates and found that natural deletions in *fadB4*, a gene associated with cell envelope biosynthesis, had arisen in the outbreak strains. In a macrophage infection model, *fadB4-*deficient strains accumulated rapidly, inducing lipid droplets and IL-1β secretion (Fig 1) [21].

Importantly, these studies not only unveil unique characteristics of *M. tuberculosis* outbreak strains but also lay the groundwork for an enhanced understanding of *M. tuberculosis* pathogenesis. By collaborating with clinicians and epidemiologists to identify unique outbreaks, researchers can focus on prominent strains with potential for elucidating novel mechanisms of *M. tuberculosis* pathogenesis. The resulting findings may extend beyond the specific strain being investigated.

## Conclusions

Whether part of a highly transmissible “modern” lineage or a geographically restricted “ancestral” lineage, *M. tuberculosis* has evolved mechanisms to resist both host immune responses and common antibiotic treatments. Epidemiological analysis of *M. tuberculosis* transmission patterns and disease characteristics, including site of disease, will be important in defining phenotypic associations. Bioinformatic analysis will continue to uncover novel variations between lineages and sub-lineages, including genetic and epigenetic modifications that impact drug susceptibility, dissemination within a host, and other characteristics unique to one or more lineages. Evolutionary analyses will help to untangle the complex evolutionary relationship between *M. tuberculosis* and humans, revealing regions of the *M. tuberculosis* genome with evidence of positive selection and convergent evolution. Finally, analysis of outbreak strains and variants in experimental models can reveal mechanisms of mycobacterial pathogenesis that may not be evident when modeling disease with standard *M. tuberculosis* laboratory strains.

*M. tuberculosis* strain identity alone does not determine TB infection outcome; the genetic diversity of host populations and a wide variety of environmental factors are among other contributors to disease [22]. However, understanding the consequences and origins of *M. tuberculosis* genomic diversity has helped to identify unappreciated molecular and cellular mechanisms that underlie the success of *M. tuberculosis* as a pathogen.
